# Does Central Monitoring Lead to Higher Quality? An Analysis of Key Risk Indicator Outcomes

**DOI:** 10.1007/s43441-022-00470-5

**Published:** 2022-10-21

**Authors:** Sylviane de Viron, Laura Trotta, William Steijn, Steve Young, Marc Buyse

**Affiliations:** 1 CluePoints S.A., Avenue Albert Einstein, 2a, 1348 Louvain-la-Neuve, Belgium; 2 CluePoints Inc., King of Prussia, USA; 3 International Drug Development Institute (IDDI), Louvain-la-Neuve, Belgium; 4 Interuniversity Institute for Biostatistics and Statistical Bioinformatics (I-BioStat), Hasselt University, Hasselt, Belgium

**Keywords:** Statistical monitoring, Central monitoring, Risk-based quality management monitoring, RBM, RBQM, Key risk indicators, KRI, Site performance

## Abstract

**Background:**

Central monitoring, which typically includes the use of key risk indicators (KRIs), aims at improving the quality of clinical research by pro-actively identifying and remediating emerging issues in the conduct of a clinical trial that may have an adverse impact on patient safety and/or the reliability of trial results. However, there has to-date been a relative lack of direct quantitative evidence published supporting the claim that central monitoring actually leads to improved quality.

**Material and Methods:**

Nine commonly used KRIs were analyzed for evidence of quality improvement using data retrieved from a large central monitoring platform. A total of 212 studies comprising 1676 sites with KRI signals were used in the analysis, representing central monitoring activity from 23 different sponsor organizations. Two quality improvement metrics were assessed for each KRI, one based on a statistical score (*p*-value) and the other based on a KRI’s observed value.

**Results:**

Both KRI quality metrics showed improvement in a vast majority of sites (82.9% for statistical score, 81.1% for observed KRI value). Additionally, the statistical score and the observed KRI values improved, respectively by 66.1% and 72.4% on average towards the study average for those sites showing improvement.

**Conclusion:**

The results of this analysis provide clear quantitative evidence supporting the hypothesis that use of KRIs in central monitoring is leading to improved quality in clinical trial conduct and associated data across participating sites.

## Introduction

For years, regulatory agencies such as FDA and EMA have required that the conduct and the progress of clinical trials be monitored to ensure patient protection and high-quality studies [1, 2]. Until recently, the primary approach to meeting this requirement included frequent visits to each investigative site by designated site monitors who manually reviewed all of the patient source data to ensure it was reliably reported to the trial sponsor—a practice known as 100% source data verification (SDV) [3–6]. However, a major revision to the ICH GCP guidance was published in 2016 which strongly encouraged the use of central monitoring to more effectively and efficiently monitor trial conduct across all sites [7]. During the peak of the COVID-19 pandemic, authorities encouraged increased use of risk-based quality management (RBQM) to replace on-site monitoring activities that were prohibited due to travel restrictions. Data suggested that centralized monitoring led to a similar effectiveness as on-site monitoring pre-COVID, therefore sponsors are expected to lean towards greater adoption of RBQM going forward [8].

Central monitoring aims to detect emerging quality-related risks proactively during a clinical trial, resulting in study team intervention to address any confirmed issues and thereby drive optimal quality outcomes. A variety of tools may be applied to support central monitoring, but the following two methods are most commonly used:

- Key risk indicators (KRIs)—Metrics that serve as indicators of risk in specific targeted areas of study conduct. Sites that deviate from an expected range of values (i.e., risk thresholds) for a given KRI are flagged as “at risk”. The risk thresholds can be discrete values or set dynamically based on a statistical comparison with the trend across all sites in the study [1, 9–12].
- Statistical data monitoring—The execution of a number of statistical tests against some or all of the patient data in a study, which are designed to identify highly atypical data patterns at sites that may represent various forms of study misconduct. The types of misconduct identified may include fraud, inaccurate recording, training issues and study equipment malfunction or miscalibration [1, 3, 9–13].

Quality tolerance limits (QTLs) as referenced in ICH E6 (R2) are also commonly implemented as part of central monitoring. These can be considered a special subset of KRIs designed to monitor critical study-level risks [7, 14].

To date there has been a relative lack of direct quantitative evidence published to help confirm that central monitoring leads to improved quality. This paper presents the results of an analysis of quality improvement metrics associated specifically with the use of KRIs as part of central monitoring.

## Materials and Methods

### Central Monitoring Solution

The CluePoints RBQM platform, which includes a central monitoring solution, was the source of the data used in this analysis. The platform was launched in 2015 and enables and supports various types of RBQM analyses including risk assessment and planning, statistical data monitoring, KRIs, QTLs, duplicate patients detection and data visualization [3–6, 13, 15].

Data are typically analyzed multiple times (e.g., monthly) within the central monitoring solution during the conduct of a study. Clinical and operational data collected from various sources may be analyzed, including electronic case report forms (eCRFs), central laboratories, electronic patient reported outcome (ePRO) and electronic clinical outcome assessment (eCOA) systems, wearable technologies, and clinical trial management system (CTMS) systems. When the statistical data monitoring or KRI analysis identifies a site that exceeds a risk alert threshold (based on a *p* value or a pre-defined threshold of clinical relevance), the system triggers the creation of a risk signal for review and follow-up by members of the study team. A risk signal typically remains open until the study team determines that it is either resolved or no longer applicable (e.g., site or study closure, inability to remediate, etc.).

### Selection of KRI Data

The quality improvement analysis focused on nine KRIs that are used across numerous sponsor organizations and studies in the central monitoring platform. These nine KRIs, described in Table 1, were considered representative for the following reasons: (a) they were used in most clinical trials, (b) they monitored a wide range of clinical and operational risks (e.g., safety, compliance, data quality and enrollment and retention), (c) the risks associated with these KRIs were due to either under- and over-reporting, and (d) these KRIs used either cumulative data (all data from the very beginning of the trial) or incremental data (only data representing a subset of the last entries).

**Table 1:** KRI definitions

Category	Label (Code)	Description	Formula	Purpose
Safety	Non-serious AE rate (AERATE)	Rate of non-serious AEs per patient visit	A/B A = # of non-serious AEs across all patients at the site B = # of patient visits conducted across all patients at the site	Identify sites that are over or under-reporting. Possible reasons: -Do not understand AE reporting requirements -Failing to solicit patients for AE's -Failing to record AE's to eCRF
Compliance	Missed assessment rate (MARATE)	Proportion of expected patient assessments that were not conducted (for identified assessments of interest)	A/B A = # of assessments across all patients at the site for which the eCRF question "Was assessment performed?" = 'N' B = # of assessments across all patients at the site for which the eCRF question "Was assessment performed?" is answered	Identify sites that are non-compliant with conduct of key assessment(s). Possible reasons: -Lack of attention, distracted, too busy, etc -Misunderstanding protocol requirements -Allowing patients to opt out -Patient(s) being non-compliant for visit schedule
	Off-schedule visit (OSVRATE)	Rate of patient visits conducted outside of allowable schedule	A/B A = # of scheduled patient visits that were conducted outside of allowable visit schedule, across all patients at the site B = # of scheduled patient visits that were conducted across all patients at the site	Identify sites that are conducting patient visits outside of the schedule permitted by the protocol. Possible reasons: -Lack of awareness of allowable schedule per protocol -Lack of attention, or lack of sensitivity to protocol requirements when scheduling visits
	Protocol deviation rate (PDRATE)	Rate of protocol deviations per patient visit	A/B A = # of protocol deviations reported for the site B = # of patient visits across all patients at the site	Identify sites that present protocol non-compliance as reflected in reported protocol deviations. Reasons may be variable
Data quality	Auto-query rate (AQRATE)	Rate of auto-queries generated per datapoint submitted (last 1000 datapoints entered only)	A/B A = # of datapoints with at least 1 auto-query among the 1000 most recently entered datapoints for the site B = # the most recently entered datapoints selected for the site (minimum is 300 and maximum is 1000 datapoints)	Identify sites that may be struggling with reliable entry of patient data into the EDC system. Possible reasons: -Too busy or distracted with other priorities—rushing through data entry process -Lack of understanding/training of EDC system and/or the protocol-specific data entry requirements -Poorly created or managed source documentation, resulting in discrepant or incomplete eCRF entry
	Query response cycle time (QRESPCT)	Average cycle time from query generation to query response (last 40 queries only)	A − B A = Query response date B = Query creation date for the 40 most recent queries for the site (Sites with less than 5 queries are not assessed)	Identify sites that are slow in responding to manually generated (i.e., Data Mgt or Site Monitor) queries. Possible reasons: -Too busy or distracted with other priorities
	eCRF visit-to-entry cycle time (V2ECT)	Average cycle time from patient visit to eCRF entry (last 60 forms entered only)	A − B A = eCRF form initial entry date B = Patient visit date For 60 most recently entered forms at the site	Identify sites that are highly delayed in entering patient visit data into the EDC system. Possible reasons: -Too busy -Lack of understanding/sensitivity to importance of timely data entry
Enrollment and retention	Early termination rate (ETRATE)	Rate of early-terminated patients per patient visit	A/B A = # of early-terminated patients at the site B = # of patient post-randomization visits performed across all patients at the site	Identify sites that are struggling to retain patients through completion of the protocol. Possible reasons: -Patient mismanagement -Patients are suffering from higher rate of adverse events, possibly due to site mismanagement
	Screen failure rate (SFRATE)	Proportion of screen-failed patients out of total screened patients	A/B A = # of screen-failed patients at the site B = # of screen-failed patients at the site and # of successfully screened patients at the site	Identify sites that are screen-failing patients at an unusually high or low rate. Possible reasons: -Misunderstanding of eligibility criteria—screening too many ineligible patients -Not properly reporting screening failures (low SF rate) -Intentionally screening ineligible patients for financial reimbursement

**Figure 1:**
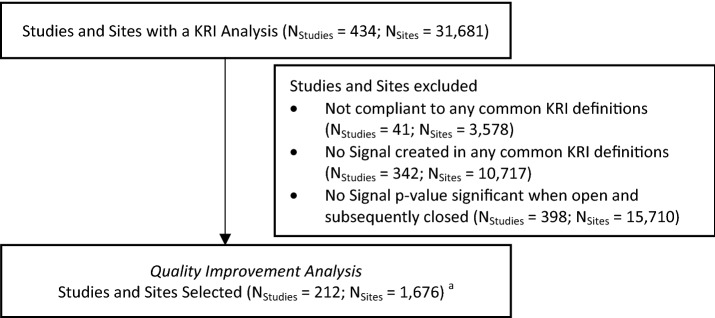
Study and sites inclusion flowchart. ^a﻿^Sites might be selected for one or multiple KRIs

The analysis was performed using data collected in the platform up to July 1st, 2022. The scope of the analysis included site-level risk signals created for the nine selected KRIs meeting the following criteria (Fig. 1):

1. The risk signal was created for a KRI that meets the common definition as described in Table 1.
2. The site’s statistical score in the system (defined as *-log*_*10*_*[p-value]*) for the KRI was > 1.3 (indicating a *p*-value < 0.05) at the time of risk signal creation.
3. The risk signal was subsequently closed by the study team.

These criteria were defined to ensure availability of evidence covering the full history of each KRI risk signal processed by the study team from initiation through final closure, and where an improvement of the statistical score was clearly expected for the site.

### Quality Improvement Analysis

The first step in the analysis was to compute the following two quality improvement metrics for each KRI risk signal:

1. KRI statistical score improvement rate—The total percent improvement in the site’s KRI statistical score (log_10_ *P-value*) from the time it was first opened (created) until it was closed by the study team. The following formula was applied:
  $$\frac{sign\left({P}_{c}\right){\mathrm{log}}_{10}\left({P}_{c}\right) -sign\left({P}_{o}\right){\mathrm{log}}_{10}\left({P}_{o}\right)}{sign\left({P}_{o}\right){\mathrm{log}}_{10}\left({P}_{o}\right)}$$
  where P_o_ is the *P*-value of the site’s KRI when the risk signal was opened.
  P_c_ is the *P*-value of the site’s KRI when the risk signal was closed.
  Sign(P_o_/P_c_) is negative (−) if the site’s observed KRI metric value is lower than the overall study trend, and positive ( +) if it is greater than or equal to the overall study trend.
2. KRI observed value improvement rate—The total percent improvement in the site’s KRI observed value relative to the overall study trend, from the time the risk signal was first opened until it was closed by the study team. The following formula was applied:
  $$\frac{\left({O}_{c}-{E}_{c}\right)-\left({O}_{o}-{E}_{o}\right)}{\left({O}_{o}-{E}_{o}\right)}$$
  where O_o_ is the site’s observed KRI metric value when the risk signal was opened.
  E_o_ is the overall study trend or “expected value” when the risk signal was opened.
  O_c_ is the site’s observed KRI metric value when the risk signal was closed.
  E_c_ is the overall study trend or “expected value” when the risk signal was closed.

## Results

In total, KRI risk signals were selected from 1676 sites across 212 studies, contributed from 23 different sponsor organizations, and comprising 11 different therapeutic areas (Table 2). A median of 2.4% of the sites were selected for the quality improvement analysis for the identified KRIs from each study (Table 3).

**Table 2:** Characteristics of the included studies

Therapeutic area	Studies N﻿ (%)	Number of patients Median [P25–P75]	Number of sites Median [P25–P75]
Cardiovascular and metabolic diseases	22 (10.4)	862 [466–1729]	70 [44–114]
Gastroenterology	5 (2.4)	404 [295–477]	94 [69–113]
Hematology	6 (2.8)	141 [62–241]	49 [31–60]
Immunology/rheumatology	25 (11.8)	408 [238–627]	61 [41–112]
Infectious disease	45 (21.2)	1446 [641–2443]	49 [26–94]
Medical aesthetics/dermatology	16 (7.5)	448 [317–1120]	75 [47–155]
Nephrology/urology	6 (2.8)	374 [314–699]	56 [34–76]
Neurology/central nervous system (CNS)	15 (7.1)	557 [203–1087]	59 [49–131]
Oncology	60 (28.3)	238 [120–655]	43 [28–109]
Pulmonary/respiratory	9 (4.2)	853 [500–1022]	83 [70–141]
Other therapeutic areas	3 (1.4)	101 [72–109]	26 [25–32]

**Table 3:** Rate of selected sites by study for each KRI

Category	KRI	Distinct studies N	Distinct study-sites N	Selected sites-KRIs by study rate (%)^a^ median [P25–P75]
**Overall**		**212**	**18,440**	**2.4 [1.3–4.3]**
Safety	AERATE	39	4305	2.6 [1.2–4.3]
Compliance	MARATE	49	4561	1.2 [0.7–2.1]
	OSVRATE	39	4654	2.1 [1.4–3.3]
	PDRATE	92	9013	2.6 [1.4–4.3]
Data quality	AQRATE	98	9225	3.8 [1.9–5.7]
	QRESPCT	58	6629	2.7 [1.7–4.2]
	V2ECT	123	12,182	2.5 [1.3–4.3]
Enrollment and retention	ETRATE	33	4487	1.9 [1.2–3.8]
	SFRATE	45	6094	1.6 [1.1–2.5]

Bold represent the aggregated results

^a^Rate % of Selected Sites-KRIs by study represents the rate of sites that fulfil the three criteria defined in the current analysis: The risk signal was created for a KRI that meets the common definition as described in Table 1. The site’s statistical score in the system (defined as *-log*_*10*_*[p-value]*) for the KRI was > 1.3 (indicating a *p*-value < 0.05) at the time of risk signal creation. The risk signal was subsequently closed by the study team

The clinical trials landscape was fairly represented, with studies selected from a broad range of therapeutic areas and study sizes (number of patients and sites). Oncology was the most frequent therapeutic area with 28.3% of studies (60/212), which included a median of 238 patients and 43 sites (Table 2). Additionally, all clinical phases were represented in the 212 studies selected from phase I (7.5%, 16/212) to phase III (56.1%, 119/212) and even phase IV or pre/post-market approval (6.6%, 14/212).

Overall, across all KRIs, quality improvement was observed in a vast majority of the risk signals—82.9% for the statistical score (1680/2027) and 81.1% for the observed KRI value (1467/1809). The percentages were similarly high for each of the nine KRIs individually (Table 4).

**Table 4:** Rate of sites and size of the data improvement

Category	KRI	Total distinct studies N	KRI Risk Signals with Improved statistical score N (%)	Statistical score improvement rate (%)^a^ median [P25–P75]	KRI risk signals with improved observed value^b^ N (%)	Observed value improvement rate (%)^c^ median [P25–P75]
**Overall**		**212**	**1680 (82.9)**	**66.1[29.8–100.0]**	**1467 (81.1)**	**72.4 [37.8–101.6]**
Safety	AERATE	39	62 (75.6)	47.9 [23.9–96.7]	58 (79.5)	23.6 [7.3–82.1]
Compliance	MARATE	49	146 (77.2)	65.6 [22.2–100]	139 (88.5)	75.2 [39.6–103.8]
	OSVRATE	39	69 (66.3)	23.1 [8.5–44.3]	68 (81.9)	28.4 [16.5–62.9]
	PDRATE	92	256 (83.9)	38.7 [19.2–63.1]	216 (76.9)	49.1 [23.6–72.1]
Data quality	AQRATE	98	364 (91.5)	83.2 [53.2–101.9]	329 (84.1)	81.0 [50.9–105.7]
	QRESPCT	58	166 (86.0)	76.5 [43.7–100.1]	132 (68.8)	77.9 [48.4–103.4]
	V2ECT	123	467 (92.1)	86.2 [53.7–102.5]	411 (84.7)	92.8 [66.6–108.2]
Enrollment and retention	ETRATE	33	78 (78.8)	33.7 [9.3–50.5]	53 (66.3)	37.4 [18.1–64.8]
	SFRATE	45	72 (73.5)	28.8 [18.7–52.4]	61 (92.4)	40.6 [26.5–66.5]

Bold represent the aggregated results

^a^Calculated only on sites with an improved statistical score

^b^Includes only sites for which the observed value changed, or number of patient visits increased from signal open to close

^c^Calculated only on sites with an improved observed value

Next, for those risk signals for which improvement was observed, the sites’ KRI statistical scores moved 66.1% closer to the expected behaviors on average, while the sites’ observed KRI values moved 72.4% closer to the expected values on average. Positive improvement was observed for each of the nine KRIs individually as well (Table 4).

These results were even stronger for data quality KRIs and more particularly for visit-to-eCRF entry cycle time (V2ECT), for which almost all sites improved (Score improved in 92.1% of the sites (467/507), Observed value improved in 84.7% of the sites (411/485)). Additionally, those sites for which V2ECT improved almost completely closed the gap towards the expected. The scores improved, on average, by 86.2% from signal open to close, and the observed value was, on average, 92.8% closer to the expected (Table 4).

**Figure 2:**
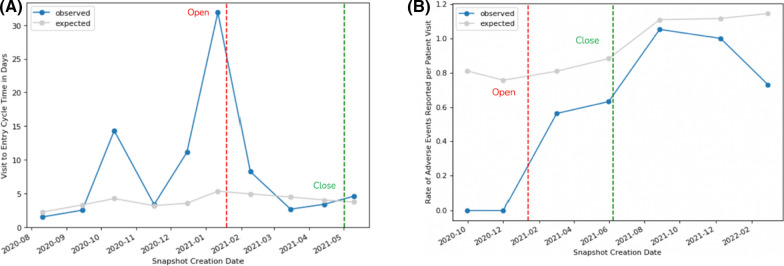
**A** V2ECT and **B** AERATE signal examples. Circles on the Observed and Expected values represent the analysis of a new increment of study data. V2ECT is calculated based on 60 most recently entered forms at the site, while AERATE takes into account cumulative data from the beginning of the trial

Figure 2 displays the evolution of observed and expected KRI values for two sample risk signals included in this analysis. Figure 2A shows the progression of a site’s V2ECT over time in an immunology trial. The signal was created after the site was observed with a significantly high V2ECT of 32 days compared to the study average of 5 days. Risk signal documentation available in the central monitoring solution revealed that the clinical research associate (CRA) followed up with the site staff to discuss their observed delay in data entry, and subsequently there was a significant improvement in their data entry timeliness. The signal was kept open by the study team for several months to confirm ongoing compliance. At the time of closure, the site’s average V2ECT (4 days) was slightly better than the study average, yielding an improvement of just over 100%.

Figure 2B shows the cumulative progression across snapshots of data of adverse event reporting rate (AERATE) over time for another site in an oncology trial. The risk signal was created in this case after the site was observed to have reported no adverse events (AEs) yet across 16 reported patient visits. The expected AERATE observed across all sites in the study was almost 0.8 AEs per patient visit, which made the site’s absence of AEs highly atypical (suspicious). Indeed, as 16 patient visits were reported, we expected an average of 12.8 AEs for the site. The risk signal documentation reported that study team representatives followed-up with and re-trained the site staff on expectations regarding AE collection and reporting. Subsequently, the site started to report AEs and part of these AEs had a start date older than the signal creation date, confirming that AEs were not reported as expected previously. The signal was closed 5 months later after the site had reported a total of 12 AEs across 19 patient visits. The site continued to improve its AE reporting behavior following closure of the signal.

## Discussion

Central monitoring tools, including KRIs, are generally designed for the purpose of continually identifying sites that are deviating from an expected pattern of quality behavior, so that study teams can intervene and address any confirmed issues [1, 2, 9]. The results of the current analysis provide clear evidence that a vast majority of the sites flagged by this approach show a significant level of improved quality related to the KRI metrics of interest.

The nine KRIs selected in the current analysis are themselves widely adopted and therefore accepted as relevant indicators of quality. Indeed, as analyzed in a TransCelerate survey, study sponsors reported that the most frequent KRIs were related to AEs, data flow, protocol deviations, treatment compliance and data quality. They also pointed out that most of the KRIs were assessed cumulatively and a lower proportion were assessed incrementally [10].

As seen in Table 4, KRIs under the “Data Quality” category (i.e., AQRATE, QRESPCT, and V2ECT) show significantly higher improvement relative to the other KRIs. This significant difference can be attributed to the way each KRI metric was designed. The three Data Quality KRIs were designed to assess sites “incrementally” based on their most recent activity (e.g., assess eCRF entry cycle time using only the 60 most recently entered eCRF forms at the site). This approach yields a more accurate measurement of the total improvement achieved since the risk signal was created. The other KRIs including AERATE were instead designed to assess sites “cumulatively” based on a full complement of their activity going back to the start of the trial. As a result, the measured improvement is artificially weighed down by inclusion of all of the site’s activity that occurred prior to creation of the risk signal. It is therefore expected that the real amount of improvement for these other KRIs was higher than we were able to measure in this analysis, and likely similar to the levels observed for the three Data Quality KRIs.

The improvement observed for the AERATE was particularly impressive. A majority (79.5%, 58/73) of the sites improved their AE reporting rate following the study team intervention. Upon review of the study team documentation on AERATE KRI risk signals, we found that in a majority of the follow-up interventions with sites, the CRAs had a discussion with the site but they were not able to confirm any issues in the site’s AE collection and reporting process. Nevertheless, the discussion with the site staff raised their awareness and led to improved AE reporting compliance.

It is important to recognize that improved quality does not come automatically through implementation of central monitoring. The degree of success achieved is highly dependent on the thoughtful design and implementation of all central monitoring tools (including KRIs) and risk follow-up processes.

To our knowledge, this is the first paper to quantitatively assess improved trial quality through the use of KRIs, as a review of the literature has not revealed any such analysis at either a study level or across studies. Currently, the literature focuses more on the correction following source data verification or on-site monitoring which have shown limited impact [12, 16, 17]. Future research work might focus on the quantitative difference in quality improvement between central monitoring and traditional study oversight methods (e.g., intensive SDV).

## Conclusion

These results provide clear evidence that central monitoring, which is recommended by regulatory agencies [1, 2]] is effective at detecting data quality issues in clinical trials. When properly implemented, managed and followed-up, KRIs enable a more targeted and efficient approach to identifying and addressing emerging quality-related risks during a study.
